# Parenting styles and externalizing problem behaviors of preschoolers: mediation through self-control abilities and emotional management skills

**DOI:** 10.3389/fpsyg.2025.1433262

**Published:** 2025-02-05

**Authors:** Zhang WenLi, Xu Tiemei, Li Shuangqi, Yu Qun, Zhu Jingbo, Sun Sijie

**Affiliations:** ^1^Normal College, Changzhou Institute of Technology, Changzhou, China; ^2^School of Educational Science, Nantong University, Nantong, China; ^3^Shanghai Normal University Tianhua College, Shanghai, China; ^4^Qinglong Central Kindergarten, Changzhou, China; ^5^Yinhai Yunfeng Kindergarten, Changzhou, China

**Keywords:** parenting styles, child psychology, preschooler, externalizing problem behaviors, emotional management, self-control, chain mediation

## Abstract

**Introduction:**

The detection rate of externalizing problem behaviors among Chinese children has been increasing year by year. Before the age of six, the problem behaviors that appear in children are predominantly externalizing problem behaviors. Family is the starting point for children's socialization. Although some studies have explored the impact of parenting styles on children's externalizing problem behaviors, only a few have explored the underlying mechanisms driving this relationship.

**Methods:**

This study attempts to fill this gap by investigating how self-control abilities and emotional management skills mediate the relationship between parenting styles and preschoolers' externalizing problem behaviors. Here, teachers and parents of 799 preschoolers from China were surveyed.

**Results:**

The findings of this investigation are 3-fold: (1) a significant association exists between the parenting styles adopted by parents and externalizing problem behaviors observed among preschoolers; (2) self-control abilities and emotional management skills independently mediate the associations between parenting styles and children's externalizing problem behaviors, highlighting their roles as mediators; and (3) sequential mediation of self-control abilities and emotional management skills elucidates a pathway through which parenting styles impact preschoolers' externalizing problem behaviors.

**Discussion:**

This study clarified the relationship between parenting styles, externalizing problem behaviors of preschoolers, self-control abilities, and emotional management skills to provide a theoretical basis for solving the externalizing problem behaviors of preschoolers.

## 1 Introduction

Externalizing problem behaviors manifest as actions that adversely affect the external environment and stem from self-regulation deficiencies, such as aggression, disciplinary problems, and Internet addiction (Goodman et al., 2010; Jin et al., 2012; Lei et al., 2019). This form of maladjustment (Dodge et al., 2003), which is detrimental to children's development and growth (Gross et al., 2009), has been widely observed. Furthermore, studies show that behaviors deemed problematic in children under 6 years primarily manifest as externalizing problems, with an increasing detection rate (from 6.4% to 17.4%) in Chinese children over the years (Basten et al., 2016; Chen et al., 2021; Wang et al., 2023). Parenting style theory underscores parents' critical role in child socialization, as they are the primary and most direct microsystem influencing early psychological and physical development (Maccoby and Martin, 1983). Although some studies have explored the impact of parenting styles on children's externalizing problem behaviors, the underlying mechanisms combining the psychosocial capacities driving this relationship in preschoolers are less studied. Through this study, we aim to gain a deeper understanding of the formation mechanism behind the externalizing problem behaviors in preschool children, offering theoretical support and practical guidance for family education and children's mental health This investigates how children's self-control and emotional management abilities mediate the relationship between parenting styles and externalizing problem behaviors of preschoolers, thereby further examining the impact and mechanisms of family factors on externalizing problem behaviors of preschoolers.

### 1.1 Parenting styles and their association with externalizing problem behaviors of preschoolers

Parenting styles embody parents' consistent behaviors and attitudes toward educating their children. Traditionally, these styles fall into three main categories: authoritative, authoritarian, and permissive (Baumrind, 1967). Permissive upbringing is not deemed suitable by Chinese parents (Wu et al., 2002). Furthermore, recent research on Chinese children indicates that a permissive parenting style does not significantly influence the problem behaviors of preschoolers, particularly aggressive behaviors (Xie and Zhang, 2024). Therefore, this study primarily focuses on the authoritative and authoritarian parenting styles.

Parenting style theory emphasizes the critical role of parenting in shaping preschoolers' developmental paths, especially in externalizing problem behaviors, a view backed by substantial research. Responsiveness and demandingness are widely accepted as two key dimensions of parenting practices (Chen et al., 2024; Garcia et al., 2024). Studies in diverse cultural settings have consistently shown that parenting styles significantly influence the development of externalizing problem behaviors in children. In particular, paternal rejection is positively correlated with children's externalizing problem behaviors (Zhang and Wang, 2022). Authoritarian parenting styles could lead to externalizing problem behaviors such as aggression, physical discomfort, and peer-social interaction (Hoeve et al., 2007; Wolfradt et al., 2003). Moreover, negative parenting psychology also affects the development of preschoolers' externalizing problem behaviors such as parenting burnout, which may lead to parental abuse and neglect of their children. Preschoolers who are abused and violent are more likely to exhibit externalizing problem behaviors, such as anger and aggression (Hansotte et al., 2021). In contrast, parental use of mindfulness can effectively reduce externalizing problem behaviors in children aged over 15 months (Park et al., 2020). At the core of authoritative parenting style is the formation of cooperative relationships between parents and their children (Holden, 2015). Effective parental support can foster children's positive development by preventing behaviors such as alcohol abuse, juvenile delinquency, and rebelliousness, which are negative forms of externalizing problem behaviors (Bean et al., 2006). Authoritative parenting style generally involves exercising appropriate behavioral control over preschool children to promote positive child development, while either insufficient (e.g., lax supervision) or excessive (e.g., harsh corporal punishment) control can lead to externalizing problem behaviors (Galambos et al., 2003).

### 1.2 Mediation of self-control abilities

Here, we explore the relationship between self-control abilities and externalizing problem behaviors in preschoolers. Self-control ability describes an individual's capacity to actively manage their psychological and behavioral processes. It encompasses the capacity to consciously choose goals, suppress impulses, resist temptations, delay gratification, and self-regulate behavior without external guidance, thus ensuring goal attainment (Inzlicht et al., 2014). Self-control, as an intra-individual factor, directly affects a child's externalizing problem behavior (King et al., 2011). Effective self-control can improve preschoolers' social adaptability and reduce their risk of drug use, emotional problems, and aggressive behaviors (Li et al., 2023; Rodríguez-Ruiz et al., 2023). In contrast, lower self-control is an important predictor of crime and a negative social life, including poor social relationships, lifestyle, and lower socioeconomic achievement (Chui and Chan, 2015; Pratt and Cullen, 2006; Evans et al., 1997). Individuals with lower self-control abilities are more prone to externalizing problem behaviors, including aggression, hyperactivity, and academic underperformance, which may escalate to smoking, drug addiction, Internet addiction, and criminal activity with age (Bunch et al., 2018; Hagger, 2013; Shepperd et al., 2015; Williamson, 2016).

Regarding the relationship between self-control abilities and parenting styles, the development of self-control ability is influenced by various factors, particularly parenting style and familial determinants (Russell et al., 2013). Self-control theory states that negative parenting practices can greatly hinder the development of a child's self-control ability (Bunch et al., 2018), parental rejection and overbearing interventions indicate deficits in children's self-control abilities. Negative parenting indirectly leads to an increase in problematic behaviors in preschoolers by reducing their level of self-control. A study of Chinese preschoolers showed that self-control plays a mediating role in the relationship between parental corporal punishment and child-externalizing problem behaviors (Hu et al., 2018). Conversely, authoritative parenting enhances a child's understanding of behavioral autonomy (Li et al., 2023), encourages trust in their judgment, and fosters self-discipline and a sense of achievement. Attachment-oriented parents who consistently monitor, acknowledge, and correct whimsical conduct enable their children to resist immediate gratification associated with adverse behaviors from an early age (Pratt and Cullen, 2006).

### 1.3 Mediation of emotional management skills

Here, we explore independent relationships among emotional management skills, parenting styles, and externalizing problem behaviors of preschoolers. Emotional management skills involve effectively recognizing, understanding, expressing, and regulating emotions to maintain them within a positive and manageable range. This skill is crucial for handling the challenges of diverse emotional experiences and adapting smoothly to societal norms (Chen, 2023). Development contextualism suggests a dynamic interaction between children's development and their ecological context (Kilica et al., 2015). Authoritative parenting positively predicts the development of children's emotional management skills, contrary to the negative effects of strict (authoritarian) or indulgent (permissive) parenting (Kilica et al., 2015). Authoritative parents help enhance preschoolers' emotional regulation through positive interactions and guidelines that are consistent with parents, which positively predict emotional management skills (Khaleque, 2013). Conversely, parents who use negation and coercive discipline hinder their children's emotional management skills (Kawabata et al., 2011). Furthermore, there is a significant correlation between children's emotional management skills and externalizing problem behaviors (Wang et al., 2009).

Recent empirical studies have highlighted the mediating role of emotional management skills in predicting parenting styles and externalizing problem behaviors in preschoolers. Children exposed to high levels of stress or strict parental control have a higher risk of emotional suppression, depression, or dysregulation. They are more likely to externalize problematic behaviors when faced with emergent situations or solving problems. Conversely, children raised by authoritative parents often develop a tendency toward positive emotional regulation (Folk et al., 2014), equipping them with constructive coping strategies for conflicts or challenges (Lissa et al., 2019; Contreras et al., 2000). These findings clarify that parenting styles provide foundational knowledge for emotional regulation, which children internalize and apply in various contexts. New studies show that positive parenting practices can improve the quality of parent-child relationships and parents' behavior management skills, followed by fewer children's emotional problems and problematic behaviors (Kjøbli et al., 2023).

### 1.4 Chain mediation of self-control abilities and emotional management skills

Based on the analysis presented, self-control abilities and emotional management skills may be key mediators between parenting styles and externalizing problem behaviors in preschoolers. Emotional management skills entail effectively expressing, regulating, and applying emotions (Goleman, 2010). Maturing self-control abilities corresponds with enhanced efficiency in managing emotions, and according to the initiation hypothesis of psychological mechanisms of self-control abilities, higher self-control abilities contribute to suppressing emotional arousal and mediating emotional expressions and impulsivity (Mischel and Ayduk, 2002). Eisenberg proved that preschoolers who have a level of control over maintaining their own positive emotions and controlling their own bad emotions are those of the same age stage (Eisenberg and Fabes, 2004). A US tracking study of 1,000 3-year-old children found that children assessed as uncontrollable at age three were relatively more likely to have temper tantrums and impulsive behaviors and had more negative emotions as adults than their peers (Eric and David, 2009). There are only a few studies on self-control abilities and emotional management skills of preschoolers in China, and these studies are on the correlation between self-control abilities and emotional regulation strategies. Empirical findings indicate that positive emotion-regulation strategies are correlated with higher self-control abilities (Wang and Li, 2010). A study on the relationship between self-control ability and emotion regulation in Chinese preschoolers showed that preschoolers with superior self-control abilities are more inclined to employ positive emotional strategies when navigating problem-solving scenarios (Anna, 2016; Yu, 2019). A significant correlation between self-control abilities and emotional management skills may be inferred based on the above results.

### 1.5 The current study

In recent years, scholars have focused on the direct and indirect effects of family parenting styles on externalizing problem behaviors of preschoolers. These studies mainly included parental factors such as parenting pressure, psychological resilience, parenting mindfulness, and emotional symptoms (Ren, 2023; Huang and Wang, 2024). However, there are only a few studies including the factors of children's own development. Existing studies focus on independent relationships among preschoolers' self-control abilities, emotional management skills, parenting styles and preschoolers' externalizing problem behaviors. Furthermore, examining the direct relationship between self-control and emotion management can help elucidate their combined impact on the psychological development of preschool children. This can offer a scientific foundation for family and educational practices aimed at correcting and reducing externalizing problem behaviors in preschool children. Therefore, including the two important psychological factors of self-control ability and emotional management skills in the study of externalizing problem behaviors in preschoolers is beneficial to reduce the occurrence of externalizing problem behaviors and focus on the psychological development of preschoolers from the perspective of parenting styles. Moreover, previous mediation studies used the same subjects to complete all survey questionnaires (i.e., parent reports). The greatest problem with a single data source is the presence of common methodological biases. Therefore, it is difficult to explore a mechanism involving multiple factors in externalizing problem behaviors of preschoolers. In summary, this study is framed within the parenting style theory, integrating developmental contextualism and self-control theories. The impacts of authoritative and authoritarian parenting styles on externalizing problem behaviors in preschoolers are examined by combining reports from parents and teachers. It also explores how preschoolers' self-control abilities and emotional management skills influence these behaviors. This study proposes the following hypotheses:

H1: Parenting styles relate to externalizing problem behaviors in preschoolers; authoritative parenting inversely correlates with externalizing problem behaviors whereas authoritarian parenting positively correlates with externalizing problem behaviors.
H2: Self-control abilities play a mediating role between parenting styles and preschoolers' externalizing problem behaviors; authoritative parenting style positively relates with the self-control abilities of preschoolers, self-control ability negatively relates with the externalizing problem behaviors of preschoolers, authoritarian parenting style negatively relates with the self-control abilities of preschoolers, and self-control ability negatively relates with the externalizing problem behavior of preschoolers.
H3: Emotional management skills of children act as a mediating factor through which parenting styles affect the emergence of externalizing problem behaviors in preschoolers. Authoritative parenting style positively relates with emotional management skills of preschoolers, which negatively predicts their externalizing problem behaviors; authoritarian parenting style negatively relates with emotional management skills of preschoolers, which negatively predicts their externalizing problem behaviors.
H4: Parenting styles may predict preschoolers' externalizing problem behaviors through the chain mediation effect of self-control abilities and emotional management skills.

**Figure 1:**
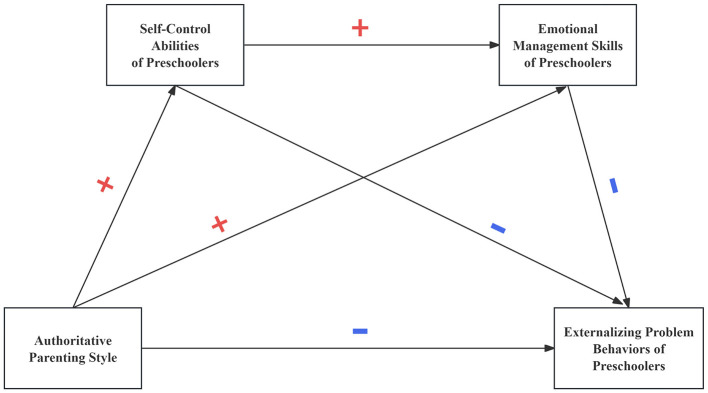
Research hypothesis model M1.

**Figure 2:**
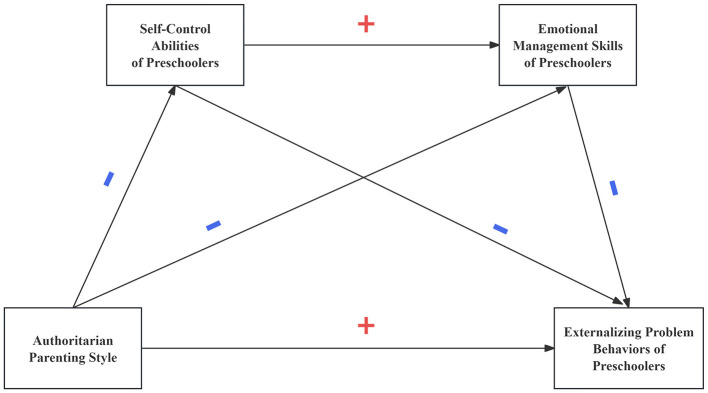
Research hypothesis model M2.

The conceptual connections among these variables are illustrated in Figures 1, 2 below:

## 2 Methodology

### 2.1 Participants

This study employed cluster sampling to select preschoolers and their parents as the research subjects. Participants were from seven kindergartens in China. The parenting styles questionnaire and children's problem behaviors questionnaire were completed by the parents. The samples were cleaned, and only those with two parents as primary caregivers were included in the survey. The children's self-control abilities and emotional management skills questionnaires were filled out by the children's classroom teachers. As the primary supervisors of children's daily lives, teachers can provide more objective and diversified observations and assessments. They observed the performance of children in terms of self-control and emotional management skills in different environments. Some parents may have filter bias and would overestimate their child's performance. Teachers and parents should fill in the child's student number when filling in the questionnaire and match the two questionnaires with the same student number when processing the data to be used as valid data. A total of 850 questionnaires were distributed, and 807 valid questionnaires were retrieved, resulting in a recovery rate of 94.94%. Invalid questionnaires (completed too quickly or with obvious patterns) were discarded, leaving 799 valid questionnaires, with an effective response rate of 94%. There were 388 boys (48.6%) and 411 girls (51.4%; M = 5.10, SD = 0.788). Out of 799, 421 questionnaires were filled out by mothers (M = 31.20, SD = 1.519), 378 questionnaires were filled out by fathers (M = 32.19, SD =2.871), and over 60% of the respondents had higher education. The questionnaires were voluntarily completed by parents and teachers, and the present study was approved by the Institutional Review Board of the first author.

### 2.2 Measurement

#### 2.2.1 Parenting styles and dimensions questionnaire (PSDQ)

This study investigated parenting styles using a simple version of PSDQ developed by Robinson and Mandleco (1995) and translated and revised by Li et al. (2012). The questionnaire has been translated into multiple languages and is widely used in many countries. After translation and revision, it is proven to have good cultural adaptability in China, and is extensively applied (Xu et al., 2022; Wang and Zhang, 2012). This questionnaire is completed by parents. The questionnaire consists of 32 questions, including three dimensions, which are authoritative, authoritarian, and permissive parenting. The reliability of permissive parenting is low in the Chinese cultural context (Wu et al., 2002; Xie and Zhang, 2024); hence, this study mainly uses the dimensions of authoritative and authoritarian parenting, with authoritative parenting consisting of 15 items and authoritarian parenting, 12 items. This questionnaire employs a Likert 5-point scoring method, from 1 (never) to 5 (always). The higher the score, the closer the parenting style used by the parents is to the corresponding type. In this study, the Cronbach's alpha coefficients for the dimensions of authoritarian and authoritative parenting were 0.971 and 0.987, respectively. The overall Cronbach's alpha of the questionnaire was 0.855. The confirmatory factor analysis (CFA) of this questionnaire showed good model fit: χ^2^*/df* = 4.664, CFI = 0.963, TLI = 0.959, RMSEA = 0.068, and SRMR = 0.0254.

#### 2.2.2 Strengths and difficulties questionnaire (SDQ)

SDQ was used to investigate children's externalizing problem behaviors (Goodman, 2001). This questionnaire is proven to suit the Chinese cultural context and completed by parents (Kou et al., 2005). The scale comprises 25 items across five dimensions. The two dimensions of conduct problem and hyperactivity/inattention are used to study externalizing problem behaviors (Huang and Wang, 2024); hence, only these two dimensions are used in this study. Each dimension includes five questions scored on a Likert scale ranging from 0 (not true) to 2 (certainly true). Higher scores indicate more severe manifestations of such behaviors. The reliability of these selected dimensions, conduct problems, and hyperactivity/inattention, were substantiated by Cronbach's alpha values of 0.791 and 0.831, respectively. The overall Cronbach's alpha coefficient for the questionnaire was 0.972. The confirmatory factor analysis (CFA) of this questionnaire showed a good model fit: χ^2^*/df* = 3.888, CFI = 0. 979, TLI = 0.969, RMSEA = 0.060, and SRMR = 0.0462.

#### 2.2.3 Self-control abilities questionnaire

The children's self-control abilities questionnaire was developed by Yang and Dong (2005). Previous studies have proven its reliability (Sun et al., 2014) and it was completed by teachers in this study. The questionnaire comprises 22 items across four dimensions: awareness, persistence, self-discipline, and self-delayed gratification. It employs a 5-point Likert scale ranging from 1 (completely disagree) to 5 (completely agree). A higher score reflects stronger self-control abilities in children. Here, the Cronbach's alpha coefficients for the four sub-scales, awareness, persistence, self-discipline, and self-delayed gratification are 0.960, 0.877, 0.952, and 0.899, respectively. The overall Cronbach's α coefficient of the questionnaire was 0.972. The confirmatory factor analysis (CFA) in this questionnaire showed good model fit: χ^2^*/df* = 3.172, CFI = 0.975, TLI = 0.971, RMSEA = 0.052, and SRMR = 0.0294.

#### 2.2.4 Emotional management skills questionnaire

Previous studies have confirmed that the emotional management questionnaire has good reliability and validity, with a Cronbach's alpha coefficient of 0.771 (Wu, 2013). In this study, it was filled out by teachers. The questionnaire contains 30 items and includes three dimensions: emotional control ability, emotional awareness ability, and emotional utilization ability, scored using a Likert 5-point scale from 1 (completely disagree) to 5 (completely agree). Higher scores indicate better emotional management skills in children. In this study, the Cronbach's alpha coefficients for the subscales of emotional control ability, emotional awareness ability, and emotional utilization ability were 0.981, 0.975, and 0.962, respectively. The overall Cronbach's α coefficient of the questionnaire was 0.955. The confirmatory factor analysis (CFA) of this questionnaire showed good model fit: χ^2^*/df* = 4.638, CFI = 0.957, TLI = 0.952, RMSEA = 0.068, and SRMR = 0.0288.

### 2.3 Research procedure

The purpose and intention of the research were explained to kindergarten leaders. They showed the content of the survey to parents and teachers, obtained their permission, and recruited the research subjects on a voluntary basis. The questionnaire was issued electronically so that it could be withdrawn any time to ensure voluntary participation. Finally, university teachers with specialized training were employed on-site to provide instructions to both parents and teachers on how to fill out the questionnaires, ensuring that the surveys were collected and checked thoroughly afterward.

### 2.4 Data processing

We used SPSS software (version 29.0) to conduct the correlational and descriptive statistical analyses. Path analysis and chain mediation effect testing were performed using Mplus software version 8.3.

## 3 Results

### 3.1 Common method bias test

This study collected data using a combined approach of parent and teacher reports, ensuring anonymity and incorporating reverse scoring for some items as procedural controls to mitigate common method bias. These measures helped ensure that the data collected were reliable and that the results were not unduly influenced by the data collection method itself. The Harman single-factor test, a statistical method used to check for common method bias, was applied through exploratory factor analysis (EFA) to all variables in the study, which included 89 items. This test is essential for identifying whether a single factor alone can account for the majority of the covariance among the measures. The results indicated that under conditions with no rotation applied, there were 11 factors with eigenvalues >1. The first factor accounted for 39.64% of the variance, which is below the critical threshold of 40%.

### 3.2 Descriptive statistics and correlation analysis

Descriptive statistics (means and standard deviations) and correlation coefficients for the variables are presented in Table 1. Prior research in China has indicated that the sex, age, and only-child status of preschoolers significantly affect the development of their problem behaviors. Therefore, in this analysis, sex, age, and only-child status were used as control variables (Bai et al., 2022). As shown in Table 1, after controlling for the sex, age, and only-child status of preschoolers: authoritative parenting showed a significant positive correlation with preschoolers' self-control abilities and emotional management skills (r = 0.488–0.636, *p* < 0.001) and a significant negative correlation with externalizing problem behaviors (r = −0.443, *p* < 0.001); authoritarian parenting was significantly negatively correlated with preschoolers' self-control abilities and emotional management skills (*r* = −0.443 to −0.497, *p* < 0.001) and positively correlated with externalizing problem behaviors (*r* = 0.628, *p* < 0.001); self-control abilities in preschoolers were significantly positively correlated with emotional management skills (*r* = 0.585, *p* < 0.001) and significantly negatively correlated with externalizing problem behaviors (r = −0.525, *p* < 0.001); emotional management skills of preschoolers were significantly negatively correlated with externalizing problem behaviors (r = −0.518, *p* < 0.001).

**Table 1:** Mean, standard deviation, and correlation coefficients among variables test of chain mediation effects.

	**M**	**SD**	**1**	**2**	**3**	**4**	**5**	**6**	**7**	**8**	**9**	**10**	**11**	**12**	**13**	**14**
1. Conscientiousness	3.612	1.040	1													
2. Persistence	3.368	0.937	0.710^***^	1												
3. Self-control	3.636	0.949	0.792^***^	0.767^***^	1											
4. Self-Delayed Gratifcation	3.484	0.884	0.701^***^	0.731^***^	0.805^***^	1										
5. Self-Control Abilities	3.545	0.870	0.917^***^	0.863^***^	0.935^***^	0.881^***^	1									
6. Emotional Perception	3.770	0.989	0.664^***^	0.634^***^	0.735^***^	0.712^***^	0.760^***^	1								
7. Emotional Utilization	3.554	0.966	0.468^***^	0.481^***^	0.509^***^	0.483^***^	0.536^***^	0.631^***^	1							
8. Emotional Control	3.159	1.167	0.111^***^	0.119^***^	0.153^***^	0.152^***^	0.146^***^	0.206^***^	0.065	1						
9. Emotional Management Skills	3.462	0.775	0.500^***^	0.495^***^	0.571^***^	0.552^***^	0.585^***^	0.759^***^	0.587^***^	0.765^***^	1					
10. Authoritarian Parenting Style	2.213	1.019	0.449^***^	−0.412^***^	−0.500^***^	−0.416^***^	−0.497^***^	−0.492^***^	−0.317^***^	−0.192^***^	−0.435^***^	1				
11. Authoritative Parenting Style	3.753	1.189	0.585^***^	0.562^***^	0.596^***^	0.544^***^	0.636^***^	0.594^***^	0.424^***^	0.161^***^	0.488^***^	−0.507^***^	1			
12. Conduct Problems	0.478	0.450	−0.354^***^	−0.378^***^	−0.441^***^	−0.409^***^	−0.435^***^	−0.447^***^	−0.257^***^	−0.199^***^	−0.403^***^	0.482^***^	−0.385^***^	1		
13. Hyperactivity/Inattention	0.787	0.499	−0.318^***^	−0.378^***^	−0.346^***^	−0.357^***^	−0.381^***^	−0.362^***^	−0.239^***^	−0.260^***^	−0.399^***^	0.488^***^	−0.304^***^	0.192^***^	1	
14. Externalizing Problem Behaviors of Preschoolers	1.265	0.729	−0.434^***^	−0.489^***^	−0.506^***^	−0.494^***^	−0.525^***^	−0.521^***^	−0.320^***^	−0.299^***^	−0.518^***^	0.628^***^	−0.443^***^	0.741^***^	0.801^***^	1

^*^p < 0.1;

^**^p < 0.05;

^***^p < 0.001.

### 3.3 Chain mediation effects test

The study used authoritative and authoritarian parenting styles as independent variables and preschoolers externalizing problem behaviors as dependent variables. The sex, age, and only-child status of the preschoolers were used as control variables. Two structural equation models, referred to as Models M1 and M2, were constructed to test the mediating effects of children's self-control abilities and emotional management skills on the relationship between the two types of parenting styles and externalizing problem behaviors in preschoolers.

#### 3.3.1 Chain mediation effect test for model M1

**Figure 3:**
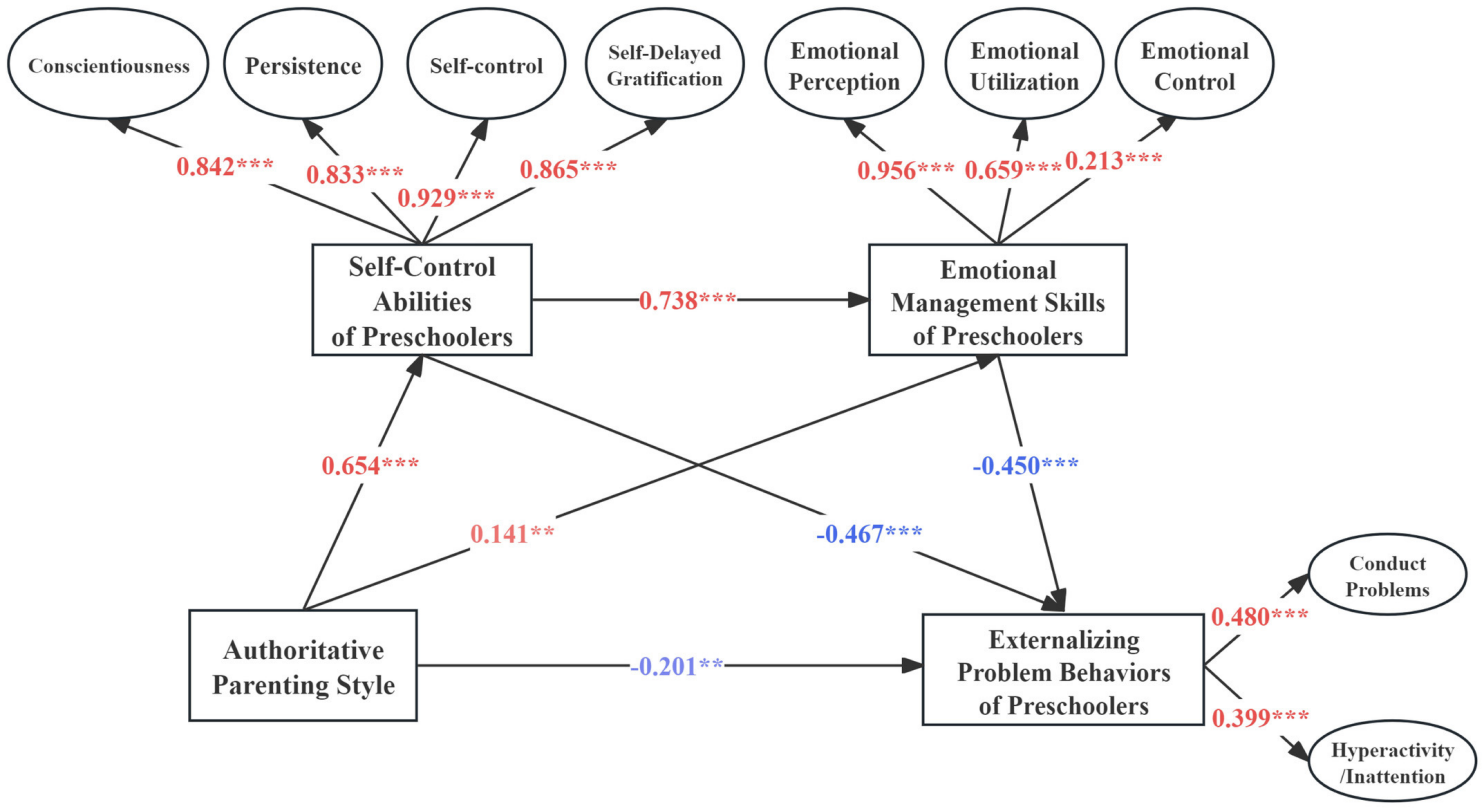
M1 chain mediation effect model. *p* < 0.1; **p < 0.05; ***p < 0.001.

Model M1, which tested the chain mediation effects of authoritative parenting style, demonstrated a good fit with the data, as shown by the following fit indices: χ^2^*/df* = 4.19, CFI = 0.979, TLI = 0.968, and RMSEA = 0.063. The factor loadings within the measurement model ranged from 0.213 to 0.956, indicating a satisfactory to excellent representation of the constructs by the observed variables, as shown in Figure 3. In terms of path relationships, authoritative parenting style positively predicted self-control abilities (β = 0.654, *p* < 0.001) and emotional management skills (β = 0.141, *p* < 0.01) of preschoolers. It also negatively predicted externalizing problem behaviors (β = −0.201, *p* < 0.01), suggesting that higher levels of authoritative parenting styles are associated with fewer externalizing problem behaviors. Furthermore, preschoolers' self-control abilities significantly positively predicted their emotional management skills (β = 0.738, *p* < 0.001) and negatively predicted externalizing problem behaviors (β = −0.467, *p* < 0.001), indicating that better self-control ability is linked to reduced problematic behaviors. Additionally, emotional management skills negatively predicted externalizing behaviors (β = −0.450, *p* < 0.001), showing that better emotional management skills are associated with fewer externalizing problem behaviors.

This study utilized the bias-corrected bootstrap method, a robust resampling technique, to analyze the pathway effects between authoritative parenting styles and externalizing problem behaviors in preschoolers. By conducting 5,000 bootstrap samples, the analysis calculated 95% confidence intervals for each pathway effect. The results shown in Table 2 confirm that the 95% confidence intervals for all evaluated pathways did not include zero, substantiating the significance of the mediation effects. Specifically, the mediation effect of self-control abilities on externalizing problem behaviors was quantified at −0.306 (*p* < 0.001, 95% CI = [−0.475 to −0.136]), while the mediation effect of emotional management skills was quantified at −0.063(*p* < 0.05, 95% CI = [−0.111 to −0.016]). Furthermore, the combined mediation effect of both self-control abilities and emotional management skills was significant at −0.217(*p* < 0.05, 95% CI = [−0.352 to −0.082]). Collectively, these three indirect pathways accounted for 74.5% of the total effect, indicating a strong mediating role of these skills in the relationship between authoritative parenting style and preschoolers' externalizing problem behaviors.

**Table 2:** Path coefficient analysis of the M1 mediation model.

**Effect**		**β**	**SE**	***t***	***P***	**LLCI**	**ULCI**	***df***
Direct effect	A → D	−0.201	0.075	−2.689^**^	0.007	−0.348	−0.055	775
Indirect effect	A → B	0.654	0.023	27.897^***^	0.000	0.608	0.700	763
	A → C	0.141	0.042	3.375^**^	0.001	0.059	0.222	755
	B → C	0.738	0.045	16.411^***^	0.000	0.650	0.826	748
	B → D	−0.467	0.134	−3.487^***^	0.000	−0.730	−0.205	768
	C → D	−0.450	0.129	−3.502^***^	0.000	−0.702	−0.198	760
	A → B → D	−0.306	0.086	−3.540^***^	0.000	−0.475	−0.136	768
	A → C → D	−0.063	0.024	−2.616^**^	0.009	−0.111	−0.016	744
	A → B → C → D	−0.217	0.069	−3.146^**^	0.002	−0.352	−0.082	723
Total effect	A → D	−0.787	0.071	−11.124^***^	0.000	−0.926	−0.648	775

A: Authoritative parenting style; B: Self-control abilities; C: Emotional management skills; D: Preschoolers' externalizing problem behaviors. LLCI refers to the lower limit of the 95% interval of the estimate and ULCI refers to the upper limit of the 95% interval of the estimate. p < 0.1;

^**^p < 0.05;

^***^p < 0.001.

#### 3.3.2 Chain mediation effect test for model M2

**Figure 4:**
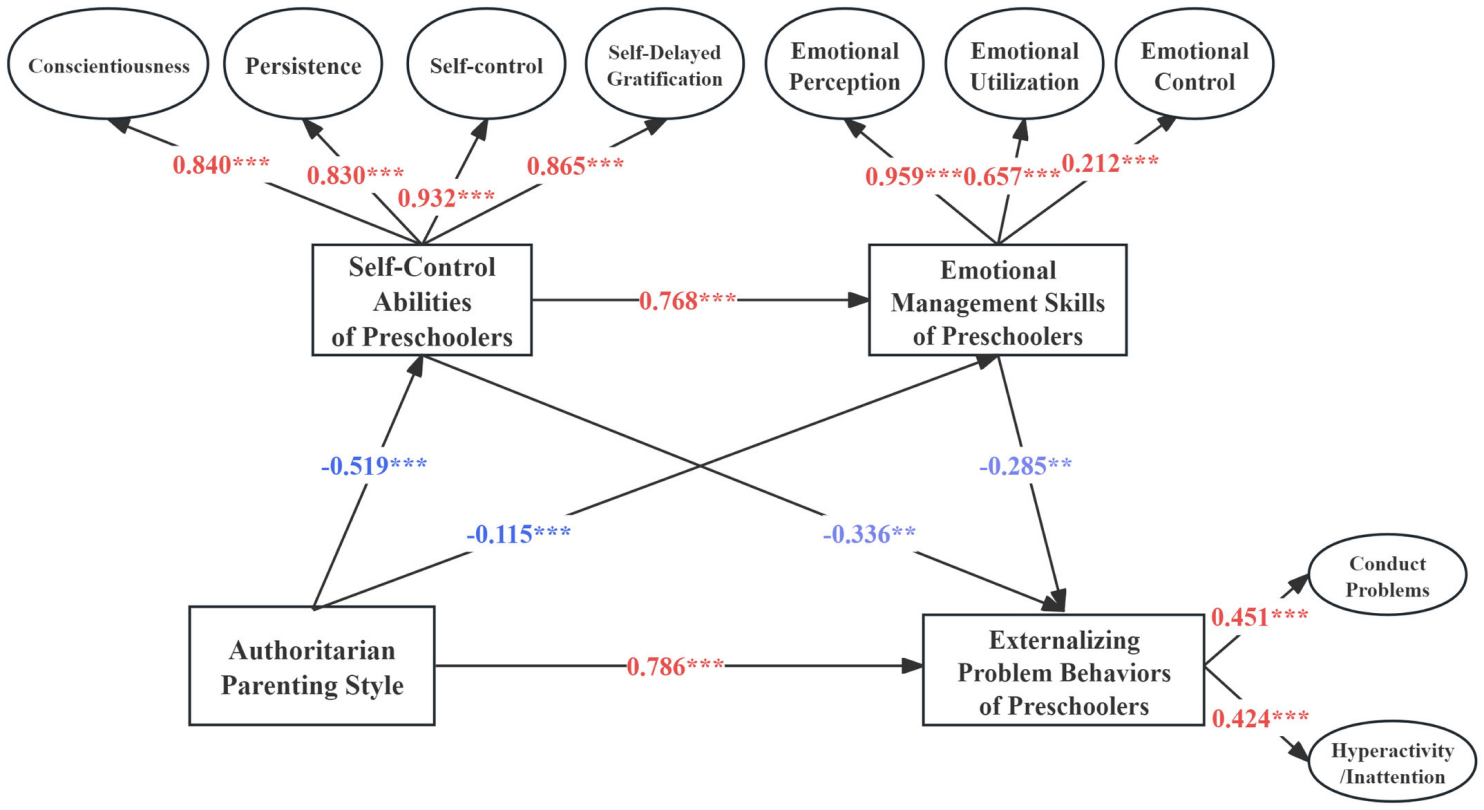
M2 chain mediation effect model. *p* < 0.1; **p < 0.05; ***p < 0.001.

The results of Model M2, which explored the chain mediation effects of authoritarian parenting style on preschoolers' externalizing problem behaviors, indicated a well-fitting multiple mediation model (χ^2^*/df* = 4.51, CFI = 0.977, TLI = 0.965, RMSEA = 0.066). Factor loadings within the measurement model ranged from 0.212 to 0.932. As illustrated in Figure 4, authoritarian parenting style negatively predicted preschoolers' self-control abilities (β = −0.519, *p* < 0.001) and emotional management skills (β = −0.115, *p* < 0.001), but positively predicted externalizing problem behaviors (β = 0.786, *p* < 0.001). Furthermore, children's self-control abilities significantly positively predicted their emotional management skills (β = 0.768, *p* < 0.001) and negatively predicted externalizing problem behaviors (β = −0.336, *p* < 0.01). Additionally, emotional management skills were found to negatively predict externalizing problem behaviors (β = −0.285, *p* < 0.01), underscoring the critical role of psychological skills in mediating the effects of parenting styles on preschoolers externalizing problem behaviors.

Using the bias-corrected bootstrap method, 5,000 resamples were conducted to analyze the pathway effects of authoritarian parenting style on preschoolers' externalizing problem behaviors, with the calculation of 95% confidence intervals for each pathway. The results, as presented in Table 3, indicate that the 95% confidence intervals for all pathways did not include zero, confirming that the mediation effects were statistically significant. Specifically, the mediation effect of self-control abilities was 0.174 (*p* < 0.05, 95% CI = [0.067–0.281]), the mediation effect of emotional management skills was 0.033 (*p* < 0.05, 95% CI = [−0.001–0.064]), and the combined mediation effect of both self-control abilities and emotional management skills was 0.114 (*p* < 0.05, 95% CI = [0.030–0.198]). All three indirect pathways were significant, with the mediation effects accounting for 29.0% of the total effect.

**Table 3:** Path coefficient analysis of the M2 mediation model.

**Effect**		**β**	**SE**	***t***	***p***	**LLCI**	**ULCI**	***df***
Direct effect	A → D	0.786	0.084	9.402^***^	0.000	0.622	0.950	778
Indirect effect	A → B	−0.519	0.034	−15.376^***^	0.000	−0.585	−0.452	766
	A → C	−0.115	0.030	−3.861^***^	0.000	−0.173	−0.057	758
	B → C	0.768	0.034	22.527^***^	0.000	0.701	0.834	748
	B → D	−0.336	0.103	−3.273^**^	0.001	−0.537	−0.135	768
	C → D	−0.285	0.104	−2.747^**^	0.006	−0.489	−0.082	760
	A → B → D	0.174	0.055	3.189^**^	0.001	0.067	0.281	756
	A → C → D	0.033	0.016	2.042^**^	0.041	0.001	0.064	748
	A → B → C → D	0.114	0.043	2.650^**^	0.008	0.030	0.198	726
Total effect	A → D	1.107	0.087	12.740^***^	0.000	0.936	1.227	778

A: Authoritarian parenting style; B: Self-control abilities; C: Emotional management skills; D: Preschoolers' externalizing problem behaviors. LLCI refers to the lower limit of the 95% interval of the estimate and ULCI refers to the upper limit of the 95% interval of the estimate. p < 0.1;

^**^p < 0.05;

^***^p < 0.001.

## 4 Discussion

This study was conducted in seven kindergartens across Jiangsu Province, China, to examine the mechanisms by which parenting styles influence the externalizing problem behaviors of preschoolers. This study found that preschoolers' self-control abilities can regulate the relationship between parenting styles and externalizing problem behaviors of preschoolers. Similarly, preschoolers' emotional management skills can influence the relationship between parenting styles and externalizing problem behaviors of preschoolers. Furthermore, this study provides an empirical basis for preschoolers' self-control abilities and emotion management skills by acting as a linkage mediator between parenting styles and externalizing problem behaviors of preschoolers. This study verifies and expands the rationality of the parenting style theory (Maccoby and Martin, 1983), psychological mechanisms of self-control abilities (Mischel and Ayduk, 2002), and developmental contextualism (Kilica et al., 2015). Our study separately examines the relationship between parenting styles (authoritative parenting and authoritarian parenting styles), preschoolers' self-control abilities, emotional management skills, and externalizing problem behaviors, providing many perspectives, enriching and revising theories on preschoolers' externalizing problem behaviors. In addition, this study confirmed for the first time that preschoolers' self-control abilities can directly and significantly affect their emotional management abilities, complementing the research gap especially in the context of Chinese research, and promoting the development of psychological-related theories in preschoolers.

### 4.1 The association between parenting styles and externalizing problem behaviors of preschoolers

This study indicates that different parenting styles can significantly predict externalizing problem behaviors among preschoolers. Authoritative parenting style is significantly negatively correlated with externalizing problem behaviors of preschoolers, while authoritarian parenting style was significantly positively correlated. This conclusion is consistent with a previous study (Park and Dotterer, 2018). However, studies on Chinese families found that authoritarian parenting style has brought some benefits (Chao, 1994, 2001). The reason for this conclusion may be the traditional Chinese parenting concept, in which traditional Chinese parents prefer to use harsh socialization strategies to discipline children to meet their parents' demands for personal development (Chui and Chan, 2015). Over the past 30 years, China's reform and opening up, rapid economic development, and renewal of educational philosophy have led more parents to adopt scientific parenting concepts to raise their children. This study found that warm parenting significantly reduces externalizing problem behaviors of preschoolers, therefore hypothesis 1 is confirmed. It extends the findings and shows that various parenting factors from both parents significantly predict externalizing problem behaviors among preschoolers. This study also provides support for ecological systems theory and developmental contextualism. Authoritative parents emphasize a balance of love and constraint, focusing on grace and authority in child-rearing (Xiang and Lei, 2021). These characteristics are subtly imitated and learned by children in daily communication, thus affecting the externalization of children's problem behaviors. Therefore, children can deal with problems calmly when they encounter problems, showing fewer externalizing problem behaviors and increasingly friendly behaviors (Alizadeh et al., 2011). Authoritarian and harsh parenting styles can temporarily suppress immediate behavioral issues in young children. However, as children internalize aggressive parental language and physical threats, they may exhibit heightened aggression, resulting in an escalation of externalizing problem behaviors (Fu et al., 2022). Our findings show that authoritative parenting style leads parents to focus on preschoolers' behavioral changes and help the preschoolers to master solving and coping strategies, positive and effective feedback, which is conducive to early detection and timely intervention of preschooler externalizing problem behaviors, and reduce the level of externalizing problem behaviors.

### 4.2 Independent mediation of self-control abilities and emotional management skills

Parenting styles can indirectly predict preschoolers' externalizing problem behaviors through children's self-control abilities. This conclusion supports previous research findings and confirms hypothesis 2 (Zhang et al., 2021). Positive parenting styles (such as authoritative style) benefit preschoolers in integrating into the family, trusting family members, and developing higher levels of self-control abilities (Shen et al., 2018). Individuals with high self-control abilities are less impulsive and can better regulate impulsive behaviors, resulting in fewer externalizing problem behaviors such as rule-breaking and aggression (Sosa and Santos, 2018). Conversely, authoritarian parenting limits children's autonomy, and authoritarian parents often tell their children what they must do rather than cultivating their autonomy. Thus, children lack a sense of security and confidence, and lose the sense of control over their behaviors. This may result in lower self-control abilities and increased impulsive behaviors (Tung et al., 2019). Studies conducted on Chinese children suggest that excessive levels of emotional warmth may weaken self-control in preschool children (Wang et al., 2024), potentially influencing the frequency of their externalizing problem behaviors. Therefore, to effectively reduce the externalizing problem behaviors of preschoolers, authoritative parents should also grasp the appropriate emotional warmth level in the process of raising children.

Emotional management skills mediate the relationship between parenting styles and externalizing problem behaviors in preschoolers. This result confirms hypothesis 3. Parents who adopt different parenting methods show different emotional management skills. Children will subtly acquire different emotional management skills in daily life. Positive emotional management skills can help children engage in behaviors that align with social norms. Parents with negative parenting styles have a more significant negative predictive effect on preschoolers' emotional management skills (Jaffe et al., 2010). Preschoolers with low emotional management skills are more likely to exhibit externalizing problem behaviors such as aggression (Sun et al., 2011). Authoritative parents are highly involved in their preschoolers' daily activities, engaging in meaningful parent-child interactions and fostering close relationships. Parents provide support, value their children's opinions, and assist in the development of emotional perceptions and expressions. These efforts aid preschoolers in developing effective emotional management skills. They also acquire foundational social skills and norms. Consequently, this proactive approach reduces the likelihood of preschoolers exhibiting externalizing problem behaviors. There are two common phenomena in authoritarian Chinese families: First, authoritarian parents tend to employ more reprimanding and punishment strategies when raising preschoolers (Bluth and Wahler, 2011). Second, they take on the role of “overseer” and control all aspects of the child's life (Hwang et al., 2023). Preschoolers who grow up in this family environment do not know how to regulate their emotions when they encounter problems; show low-level emotional management skills, such as irritability and anger; and externalize the behavior of getting along with people and aggressive problem behaviors. When expressing these negative emotions, they often exhibit externalizing problem behaviors, such as aggression and destructiveness (Gao et al., 2023).

### 4.3 Chain mediation effects of self-control abilities and emotional management skills

Parenting styles influence preschoolers' externalizing problem behaviors through a sequential mediation pathway. This pathway involves self-control abilities and emotional management skills. Hypothesis 4 is confirmed, and this finding helps increase research focus on the influencing factors of externalizing problem behaviors in preschoolers, especially focus on internal psychological factors. Authoritative parenting emphasizes nurturing children in a positive and warm environment. When parents exhibit more positive emotions and warmth during interactions with their children, their self-control abilities improve. Conversely, when authoritarian parents show excessively negative emotions such as aggression and authoritarianism, children's self-control abilities may be reduced (Blair et al., 2014). According to developmental contextualism, individual development follows a cyclical pattern (Blair et al., 2014; Lerner, 2007). Children with high self-control abilities significantly outperform their peers in maintaining positive emotions and controlling negative ones (Eisenberg and Fabes, 2004). Our study is the first to empirically demonstrate that self-control ability can directly and positively affect emotional management ability. This implies that high levels of self-control abilities positively predict emotional management skills. Children with positive emotional management skills can use correct emotional management strategies to solve problems, and emotional management skills can reduce the occurrence of externalizing problem behaviors (Morris et al., 2007).

### 4.4 Implication

This study focuses on externalizing problem behaviors among Chinese preschoolers. This study has important theoretical implications. First, parenting styles predict preschoolers' externalizing problem behaviors. Self-control abilities and emotional management skills have independent and chain meditation functions between parenting styles and externalizing problem behaviors in preschoolers. This study clarifies the relationships among four variables: parenting style, preschoolers' self-control abilities, preschoolers' emotional management skills, and preschoolers' externalizing problem behaviors. This study is the first to confirm that the self-control ability of preschoolers can directly and significantly affect their emotional management abilities. Second, this study is based on parenting style theory, psychological mechanisms of self-control abilities, and developmental contextualism to dissect the roles of internal self-control abilities and emotional management skills in mediating the relationship between parenting styles and externalizing problem behaviors. This approach validates the relevance of these theoretical frameworks within the context of Chinese preschoolers and offers valuable perspectives for fostering their wellbeing.

This study has important practical significance in reducing the externalizing problem behaviors of preschoolers and promoting their scientific development. This study found that authoritative parenting style significantly and negatively predicted the occurrence of externalizing problem behaviors in preschoolers. First, it encourages parents of preschoolers to focus on concepts and methods of authoritative parenting. Second, by offering high levels of support and responsiveness in everyday parenting and creating a warm, nurturing environment, it is possible to foster the development of children's self-control abilities and emotional management skills, thereby reducing the occurrence of externalizing problem behaviors among preschoolers. Final, the results suggest that educators and policymakers should concentrate on the role of family factors and psychological development in children. They should also advocate a positive and supportive parenting style to promote children's physical and mental health and social adaptability.

### 4.5 Limitations and future directions

Our study has some limitations. First, owing to the selection of research tools and limited objective conditions, only authoritative and authoritarian parenting methods were studied; neglected and indulgent parenting methods were not included. A recent study in China found differences between four parenting methods (Chen et al., 2024), and future studies are necessary to examine the influence of these four parenting styles on externalizing problem behaviors in preschool children. Second, this study used a cross-sectional research design to explore the association between parenting styles and preschoolers' externalizing problem behaviors, which cannot determine the causality between the variables. Future studies could employ longitudinal study designs and other research designs to examine the directionality among the variables. Third, this study only explored the factors within the family system that affect the externalizing problem behaviors of preschoolers and did not involve other environmental systems such as kindergartens and communities. Future studies should explore the related external factors affecting the externalizing problem behaviors of preschoolers and their mechanisms. Final, the subjects of this study were mainly from Eastern China, and it has not been confirmed whether different cultural backgrounds and geographical environmental factors affect the conclusions of this study. Therefore, scholars should conduct studies with larger and more diverse sample sizes to generalize the findings to different countries and regions.

## 5 Conclusion

This study investigated the effects of two different types of parenting styles on preschoolers' externalizing problem behaviors and explored the mediating roles of self-control abilities and emotion management skills. This study found that parenting styles significantly affect preschoolers' externalizing problem behaviors, with self-control abilities and emotional management skills mediating the impact of parenting styles on preschoolers' externalizing problem behaviors. Furthermore, the study discovered that self-control abilities and emotional management skills act as chain mediators between parenting styles and preschoolers' externalizing problem behaviors. This study verifies and expands the rationality of parenting style theory (Maccoby and Martin, 1983), psychological mechanisms of self-control abilities (Mischel and Ayduk, 2002), and developmental contextualism (Kilica et al., 2015) and refines the theoretical framework of the relationship between family upbringing and preschoolers' externalizing problem behaviors. Additionally, our study recommends that families adopt authoritative parenting styles when raising preschool children, focusing on internal psychological factors (such as self-control abilities and emotional management skills). This approach helps decrease the occurrence of externalizing problem behaviors and lays a good foundation for the subsequent behavioral development of individuals.

## Data Availability

The original contributions presented in the study are included in the article/supplementary material, further inquiries can be directed to the corresponding author/s.
